# Genetic analysis of Black Tiger shrimp (*Penaeus monodon)* across its natural distribution range reveals more recent colonization of Fiji and other South Pacific islands

**DOI:** 10.1002/ece3.316

**Published:** 2012-07-22

**Authors:** Salote S Waqairatu, Leanne Dierens, Jeff A Cowley, Tom J Dixon, Karyn N Johnson, Andrew C Barnes, Yutao Li

**Affiliations:** 1 CSIRO Food Futures National Research Flagship, CSIRO Livestock Industries, Queensland Biosciences PrecinctSt. Lucia, QLD, 4067, Australia; 2 School of Biological Sciences, The University of QueenslandSt. Lucia, QLD, 4072, Australia

**Keywords:** Dispersal, microsatellite, mitochondrial DNA, *Penaeus monodon*, South Pacific islands

## Abstract

The Black Tiger shrimp (*Penaeus monodon*) has a natural distribution range from East Africa to the South Pacific Islands. Although previous studies of Indo-Pacific *P. monodon* have found populations from the Indian Ocean and Australasia to differ genetically, their relatedness to South Pacific shrimp remains unknown. To address this, polymorphisms at eight shared microsatellite loci and haplotypes in a 418-bp mtDNA-CR (control region) sequence were examined across 682 *P. monodon* from locations spread widely across its natural range, including the South Pacific islands of Fiji, Palau, and Papua New Guinea (PNG). Observed microsatellite heterozygosities of 0.82–0.91, allele richness of 6.85–9.69, and significant mtDNA-CR haplotype variation indicated high levels of genetic diversity among the South Pacific shrimp. Analysis of microsatellite genotypes using a Bayesian STRUCTURE method segregated Indo-Pacific *P. monodon* into eight distinct clades, with Palau and PNG shrimp clustering among others from Southeast Asia and eastern Australia, respectively, and Fiji shrimp clustering as a distinct group. Phylogenetic analyses of mtDNA-CR haplotypes delineated shrimp into three groupings, with shrimp from Fiji again being distinct by sharing no haplotypes with other populations. Depending on regional location, the genetic structures and substructures identified from the genotyping and mtDNA-CR haplotype phylogeny could be explained by Metapopulation and/or Member–Vagrant type evolutionary processes. Neutrality tests of mutation-drift equilibrium and estimation of the time since population expansion supported a hypothesis that South Pacific *P. monodon* were colonized from Southeast Asia and eastern Australia during the Pleistocene period over 60,000 years ago when land bridges were more expansive and linked these regions more closely.

## Introduction

The Indo-Pacific region possesses high biodiversity and numerous studies have investigated the phylogeography and genetic diversity of resident species including butterflies (Mueller and Beheregaray 2010), marine sponges (Poeppe et al. 2010), reef fish (Drew et al. 2010; Thacker et al. 2010; Winters et al. 2010), moray eels (Reece et al. 2010), and sting rays (Kuchta and Caira 2010). Depending on the species, mitochondrial (mt)DNA sequence variations showed some, such as moray eels (Reece et al. 2010), to be genetically homogeneous throughout the Indo-Pacific, whereas others such as the parrotfish *Scarus psittacus* (Winters et al. 2010) showed significant genetic distinctions in individuals residing at the peripheries of its Indian and Pacific Ocean distribution range. Despite these findings, none of the studies have hypothesized any evolutionary model(s) to explain underlining mechanisms for genetic structures/substructures of these species.

To date two evolutionary models, the Member–Vagrant and Metapopulation models, have been proposed as potential means by which species have evolved. In the Member–Vagrant model, precise species homing restricts gene flow resulting in locally adapted gene pools with stable genetic structures, and thus with clear isolation-by-distance (ID) geographic organizations (Sinclair 1988; Garant et al. 2000; Ensing et al. 2011). In the Metapopulation model, local species subpopulations are linked through gene flows and experience recurring extinction–recolonization events due to environmental instability (Hanski 1998; Ensing et al. 2011).

**Figure 1:**
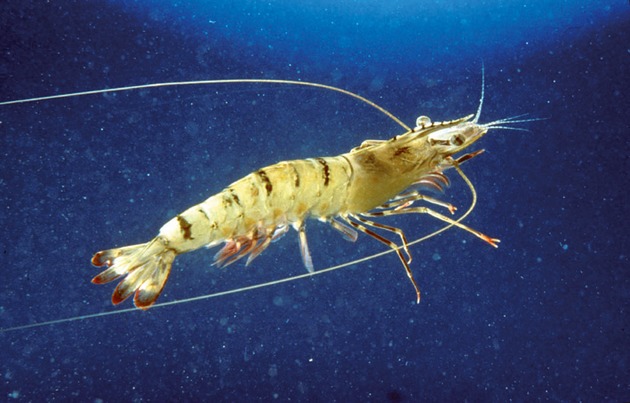
Photograph of a Black Tiger shrimp (*Penaeus monodon*), a species distributed and cultured widely across the Indo-Pacific. Image courtesy CSIRO Marine and Atmospheric Research.

The Black Tiger shrimp (*Penaeus monodon*) (Fig. 1) has a natural Indo-Pacific distribution range and is an important aquaculture species across this region. Despite its economic importance, however, there is very limited information on the evolution and colonization of *P. Monodon* in the Indo-Pacific region. Most genetic studies have been limited to *P. monodon* indigenous to regions including Australia (Mulley and Latter 1980; Benzie et al. 1992, 1993; Brooker et al. 2000), Thailand (Tassanakajon et al. 1997; Supungul et al. 2000; Klinbunga et al. 2001), India (Kumar et al. 2007), Indonesia (Sugama et al. 2002), the Philippines (Xu et al. 2001), and South Africa (Forbes et al. 1999). A few studies have examined genetic diversity between major populations distributed more widely such as eastern Africa, Southeast Asia and Australia (Bouchon et al. 1994; Duda and Palumbi 1999; Benzie et al. 2002; You et al. 2008). From restriction fragment length polymorphism (RFLP) analyses of mitochondrial DNA (mtDNA) sequence diversity across Indo-West Pacific populations, Benzie et al. (2002) showed Australasian and Indian Ocean populations to be genetically distinct. By examining differences in intron sequences in the gene encoding elongation factor-1*α*, Duda and Palumbi (1999) identified similar genetic distinctions. More recently, a study of microsatellite loci polymorphisms combined with mtDNA control region (CR) sequence differences also found *P. monodon* indigenous to regions west of India to be dissimilar to those indigenous to western Pacific Ocean regions (You et al. 2008).

Although *P. monodon* from Indian Ocean, Southeast Asian, and Australian regions have been examined in some detail, nothing is yet known about the genetic structure of *P. monodon* indigenous to South Pacific Island regions, the eastern boundary of its natural range, and when and how these regions were colonized. In addition, few attempts have been made to relate data on regional genetic divergences to how *P. monodon* have evolved and became distributed across the entire Indo-Pacific region.

In an attempt to resolve such questions, here we examined *P. monodon* from Fiji, Palau, and Papua New Guinea (PNG) as well as 14 other locations distributed widely across the Indo-Pacific. To examine genetic diversity, phylogenetic analyses were undertaken on a 418-nucleotide (nt) mtDNA-CR sequence and two multiplexed polymerase chain reaction (PCR) tests adapted from tests described previously (Li et al. 2007; Dixon et al. 2008) were used to identify heterozygosity across 11 microsatellite loci. These analyses showed shrimp from Fiji to be genetically distinct, but shrimp from Palau and PNG to be more similar to shrimp indigenous to Southeast Asia and eastern Australia, respectively. The data supported a hypothesis that the South Pacific Islands were most likely colonized by *P. monodon* from neighboring regions to the west during the Pleistocene period over 60,000 years ago, a time when land bridges were more expansive and linked these regions more closely, thus permitting coastal migration between regions now separated by large water masses. More broadly, genetic structures and substructures observed within and between distinct populations were explainable by Member–Vagrant and Metapopulation evolutionary models, and by likely shrimp translocations across regions due to more recent aquaculture activities.

## Materials and Methods

### Sample collection and DNA extraction

**Figure 2:**
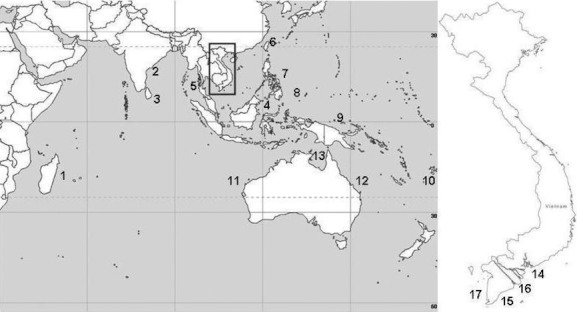
Map showing the 17 localities across the Indo-Pacific from which *Penaeus monodon* were collected. Madagascar (1), India (2), Sri Lanka (3), Brunei (4), Thailand (5), Taiwan (6), Philippines (7), Palau (8), PNG (9), Fiji (10), Western Australia (11), eastern Australia (12), northern Australia (13,) and the four Vietnam provinces (see insert) of Ben Tre (14), Bac Lieu (15), Can Tho (16), and Ca Mau (17).

A total of 682 *P. monodon* samples were collected from 17 geographic localities across the Indo-Pacific (Fig. 2), including Madagascar (*n* = 16), India (*n* = 23), Sri Lanka (*n* = 32), Brunei (*n* = 24), Thailand (*n* = 60), Taiwan (*n* = 33), the Philippines (*n* = 50), Palau (*n* = 50), PNG (*n* = 20), and Fiji (*n* = 130, originating from two east coast locations and one west coast location). Shrimp from Australia were sourced from western (*n* = 10), eastern (*n* = 50), and northern (*n* = 40) regions and shrimp from Vietnam were sourced from Ca Mau (CM, *n* = 36), Can Tho (CT, *n* = 36), Ben Tre (BT, *n* = 36), Bac Lieu (BL, *n* = 36) Provinces. All *P. monodon* were sourced from the wild except those from Vietnam, which were hatchery stocks of postlarvae spawned from wild broodstock.

Pleopods, whole postlarvae, and hepatopancreas samples were received either preserved in 95% ethanol (or RNAlater) or as extracted DNA. DNA was extracted from tissue samples using the QIAamp DNA Mini kit (QIAGEN Pty Ltd, Melbourne, Vic., Australia) as described in the kit instruction manual and stored at −20°C until used.

### Microsatellite PCR and marker assessment

Each DNA sample was genotyped using two microsatellite marker panels adapted from the panels described previously (Li et al. 2007; Dixon et al. 2008). However, instead of using all 13 markers divided into two multiplexed PCR tests, the tests were modified to employ 10 of these markers in addition to another marker DTLPm313 (Wuthisuthimethavee et al. 2003) shown to be useful for genotyping (Jerry et al. 2006) (Table S1). The two modified multiplexed PCR tests included primers for loci Pm2D2, Pm4CA, Pm8, Pm14, and DTLPm313 in System 1 and primers for loci Pm6, Pm8A2, Pm9, Pm9GG, Pm11AH, and Pm16 in System 2 (Table S1). All 11 microsatellite loci comprised either tri- or tetra-nucleotide repeats.

All PCRs were undertaken in 96-well plates using a BIO-RAD Peltier Thermal Cycler. Each reaction (10 *μ*l) containing 40 ng DNA, 5.0 *μ*L 2× Multiplex PCR Master Mix (QIAGEN), 1.0 *μ*L 10× primer mixture, and 2.0 *μ*L 5× Q buffer (QIAGEN). Each of the two 10× PCR primer mixtures contained concentrations of each primer pair optimized to reliably amplify a product. The thermal cycling conditions were 95°C for 15 min, 40 cycles of 96°C for 30 s, 60°C for 90 s, and 72°C for 1 min, followed by 60°C for 30 min to ensure complete adenylation of PCR products. All reaction sets included previously validated positive and template-free negative controls.

An aliquot (1.0 *μ*l) of each PCR using either the System 1 or System 2 primers was diluted in 100 *μ*L or 150 *μ*L molecular-grade water, respectively, followed by the addition of 10 *μ*L HiDi-formamide and 0.25 *μ*L 1200Liz size standard (Applied Biosystems, Life Technologies, Mulgrave, Vic., Australia). After heating at 96°C for 3 min, genotyping was performed using a 3130*xl* Genetic Analyzer (Applied Biosystems).

### Microsatellite genotyping data analysis

Microsatellite alleles were scored using GeneMapper version 4.0 (Applied Biosystems). For confirmation, any ambiguous alleles were scored twice independently. To assess intra-population genetic diversity, the number of alleles (*A*), the number of private alleles per loci (*Ap*), the allelic richness after correcting for differences in sample size (*Ar*), the expected heterozygosity (*H*_*E*_) in comparison to the observed heterozygosity (*H*_*o*_) (Nei 1978), and the inbreeding coefficient (*F*_*IS*_) using Fstat version 2.9.3 (Goudet 1995) were determined for the eight shared microsatellite loci examined. Genepop version 4.0 (Raymond and Rousset 1995) was used to examine if the observed genotype frequencies of individual loci deviated significantly from expected Hardy–Weinberg equilibrium (HWE) within a population, and to globally test heterozygote deficit across loci and across populations. The program was also used to test for linkage disequilibrium between loci across all 17 populations.

To investigate inter-population structures, the genetic distance (pair-wise F_ST_ value) between any two populations was calculated from allele frequency comparisons (Wright 1951). The statistical significance of an F_ST_ value was determined by analysis of 10,000 data permutations using Fstat version 2.9.3.2. To visually display the relationships between populations, a Neighbor-Joining tree was constructed using a matrix of F_ST_ values. To determine the possible number of population clusters (K) without considering sampling locations, Bayesian clustering analysis was performed using the Admixture Model of ancestry and correlated allele frequency parameters in STRUCTURE version 2.3 (Pritchard et al. 2000). The analysis used a burn-in period of 100,000 steps to minimize the effect of the starting configuration and 100,000 runs of simulation to obtain accurate parameter estimations. Tested K values ranged from 1 to *n* + 3 (*n* = 17 = number of study populations) and for each value of K, five randomized data sets were generated. K was determined by plotting the posterior probabilities of 20 values examined. Comparing posterior probabilities predicted for each K value, the value immediately below the posterior probabilities values with the smallest differential was taken as the most likely number of population clusters.

### mtDNA control region PCR

A mtDNA-CR sequence was amplified successfully with DNA from a total of 369 shrimp (Madagascar [*n* = 12], India [*n* = 16], Sri Lanka [*n* = 16], Brunei [*n* = 14], Thailand [*n* = 40], Vietnam-CM [*n* = 15], Vietnam-CT [*n* = 9], Vietnam-BT [*n* = 11], Vietnam-BL [*n* = 10], Taiwan [*n* = 23], the Philippines [*n* = 14], Western Australia [*n* = 1], eastern Australia [*n* = 25], northern Australia [*n* = 31], Palau [*n* = 33], PNG [*n* = 19], and Fiji [*n* = 81]) using the PCR primers described by Chu et al. (2003). Briefly, each PCR (25 *μ*L) contained 1× PCR buffer (Fisher Biotec, Wembley, WA, Australia), 1.5 mM MgCl_2_ (Fisher), 5U *Tth plus DNA* polymerase (Fisher), 200 nM each dNTP (Promega, Alexdrandria, NSW, Australia), 2 *μ*M each forward and reverse primer, and 100–130 ng DNA template. Thermal cycling conditions were 95°C for 3 min, 35 cycles of 94°C for 45 s, 50°C for 1 min, and 72°C for 1 min, followed by 72°C for 5 min. An aliquot (5 *μ*L) of each PCR was run in a 1.5% agarose gel to select samples containing sufficient DNA of the expected size for sequence analysis. An aliquot (5 *μ*L) of each DNA (20 *μ*L) purified using the CleanSeq method (Agencourt) was sequenced in both forward and reverse directions in 12 *μ*L reactions according to the recommended protocol for BigDye Terminator V3.1 (Applied Biosystems). DNA was sequenced using a 3130*xl* Genetic Analyzer (Applied Biosystems) and a 418 nt region internal to the PCR primers was selected to undertake phylogenetic analyses.

### Phylogenetic analysis

Each mtDNA-CR sequence chromatogram was checked, edited manually, and trimmed to 418 nt in length before being aligned using Sequencher version 4.7 (Gene Codes Corporation, Ann Arbor, MI) and ClustalX 2.0.12 (Larkin 2007). The Arlequin ver 3.11 software (Excoffier et al. 2005) was used to (1) quantify the number of haplotypes (N_*h*_), haplotype diversity (*h*), and nucleotide diversity (π) within each population, (2) quantify the levels of genetic differentiation (Ø_ST_) among populations based on Analysis of Molecular Variance (AMOVA) and (3) derive demographic evolutionary patterns based on neutrality tests for mutation-drift equilibrium as well as mismatch distribution analysis examining differences among pairs of mtDNA-CR haplotypes. Fu's F_S_ test (Fu 1997) and Tajima's D test, each using 10,000 data set permutations, were employed to check for departure from neutrality and specifically for population expansion. Mismatch distribution analysis was carried out to identify possible evidence of demographic expansions displayed by a uni-modal distribution. The Arlequin version 3.11 software was also used to perform a Mantel test to determine whether or not the levels of genetic differentiation (Ø_ST_) between populations correlated with the geographical distance between the same populations. Analysis of 1000 data permutations was employed to determine the statistical significance of correlations identified by the Mantel test.

The equation T = τ/2u was used to calculate the time since last population expansion (T), where τ is the age of the demographic change in mutation units estimated in the mismatch distribution (Rogers and Harpending 1992) and u is calculated as u = 2*μ*k, where k is the number of nucleotides assayed, and the mutation rate per nucleotide per year (*μ*) was assumed to be the average calculated for mtDNA (5.7 × 10^−8^) (Li and Graur 1991) as a universal mutation rate for the CR in crustacean mtDNA sequences is not yet available. To illustrate the genetic relationships between shrimp groups, MEGA version 4 (Tamura et al. 2007) was used to generate a phylogenetic tree using the Neighbor-Joining algorithm and Kimura-2 Parameter model of nucleotide substitution.

## Results

### Preliminary assessment of microsatellite loci

Using two multiplexed PCR tests modified from tests described previously (Li et al. 2007; Dixon et al. 2008), DNA from 615 of the 682 *P. monodon* samples from the 17 locations were genotyped successfully at eight of the 11 microsatellite loci targeted by the tests (Table S1). The PCR primer pair for locus DTLPm313 did not amplify a product with any of 20 shrimp sourced from Brunei and the PCR primer pairs for loci Pm6 and Pm14 failed to amplify products with any of 16 shrimp sourced from Madagascar. To compare all individuals from all populations rigorously, genetic analyses were restricted to the eight microsatellite loci for which data were generated universally.

### Genetic diversity determined using eight microsatellite loci

Across the 615 *P. monodon* genotyped, a high degree of allelic polymorphism was evident at each of the eight microsatellite loci examined, with a total of 459 distinct alleles being identified across all loci (Table S1). Locus Pm2D2 (81 alleles) displayed the most polymorphism followed by loci Pm16 (61 alleles), Pm11AH (58 alleles), and Pm6 (50 alleles). Locus Pm8 (18 alleles) displayed the least polymorphism. When allelic polymorphism across all eight loci was considered collectively, the mean number of alleles (*A*) per locus varied from a high of 20.1 among the 32 shrimp from Sri Lanka to a low of 6.1 among the 22 shrimp from Vietnam-CM.

Within each of the geographic groupings of shrimp, mean observed allelic heterozygosity (*H*_*o*_) values determined across all eight microsatellite loci ranged from 0.90 for the 50 shrimp from Palau down to 0.74 for the 16 shrimp from Madagascar (see Table S1). When allele numbers across all eight loci were adjusted for differences in the number of shrimp in each population, the resulting allelic richness (A_r_) values were the highest for shrimp from Sri Lanka and India (11.3) followed by shrimp from Thailand (10.4) and Palau (9.7), and were the lowest for shrimp from Vietnam-CM (5.2). Shrimp from Thailand possessed the most (26) private alleles (A_p_) unique to a population followed by shrimp from northern Australia (23) and the least (1) was possessed by the shrimp from Vietnam-CM.

In comparisons of inbreeding coefficient (F_IS_) values calculated for each of the eight microsatellite loci in shrimp from each of the 17 populations, only locus Pm8 showed no statistically significant (*P* < 0.05) departure from HWE (Table S1). Among the other seven loci, however, significant departures from HWE occurred in 30 of the 119 comparisons across the 17 populations. In HW Global tests to quantify heterozygosity levels in shrimp from each of the 17 populations, 13 of these generated positive F_IS_ values indicative of the shrimp being closely related. In contrast, shrimp from northern Australia, the Philippines, Vietnam-CM and Vietnam-BT generated negative F_IS_ values indicative of them being genetically diverse. Linkage disequilibrium tests determined from pair-wise marker analysis within each of the different populations provided evidence for some microsatellite loci being linked. In descending order, numbers of linked microsatellite loci pairs identified among shrimp from the various populations were Vietnam-BL (50), Vietnam-CT (45), northern Australia (41), the Philippines (34), Vietnam-BT (28), Sri Lanka (24), Vietnam-CM (15), Brunei (8), Taiwan (5), eastern Australia (3), Fiji (2), Palau (2), and PNG (1).

**Figure 3:**
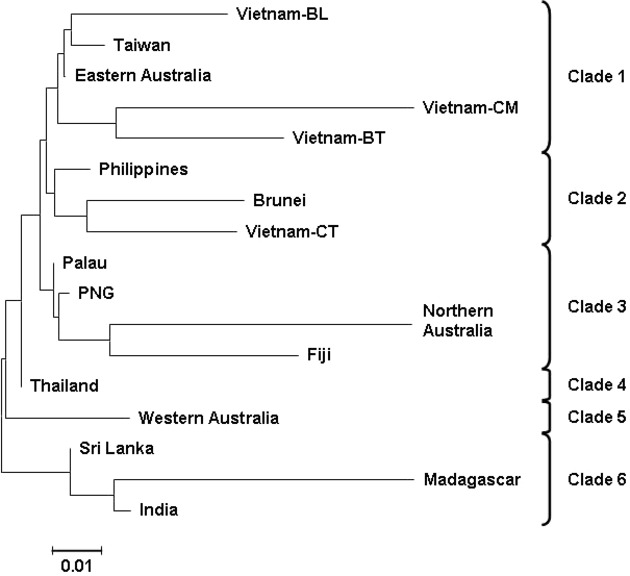
Neighbor-joining phylogenetic tree constructed from microsatellite F_ST_ values showing *Penaeus monodon* from the 17 Indo-Pacific locations to cluster within six distinct clades.

Based on analyses of the frequencies at which discrete alleles occurred across all eight microsatellite loci, the genetic distance between two populations (measured by pair-wise F_ST_ values) across the 17 populations identified statistically significant differences (*P* < 0.05) between all except eight population pairs (Table 1). As the pair-wise F_ST_ value for shrimp from eastern and western islands (∼130 km apart) of Fiji was extremely low (0.006 across the eight microsatellite loci) and not significant statistically, these Fiji shrimp were considered as a single group (data not shown). Other location pairs for which pair-wise F_ST_ values were not significant included India–Sri Lanka (0.001), Palau–Thailand (0.003), Palau–PNG (0.004), PNG–eastern Australia (0.005), PNG–Thailand (0.006), Taiwan–eastern Australia (0.009), Thailand–Western Australia (0.028), and Thailand–Vietnam-BT (0.050). In comparison to shrimp from all other populations, the most significant F_ST_ values were obtained for shrimp from northern Australia (0.067–0.178), Vietnam-CM (0.071–0.178), Madagascar (0.071–0.163), and Fiji (0.051–0.154). A Neighbor-Joining tree generated from the pair-wise F_ST_ data clustered *P. monodon* from the 17 locations into three clades comprising shrimp originating only from Pacific Ocean locations and three clades comprising shrimp predominantly from Indian Ocean locations (Fig. 3). Clade 1 comprised a node that included shrimp from eastern Australia and two sub-branches each split to include shrimp from either Vietnam-BL/Taiwan or Vietnam-CM/Vietnam-BT. Clade 2 included a branch comprising shrimp from the Philippines and a branch split to comprise shrimp from either Brunei or Vietnam-CT. Clade 3 included branches comprising shrimp from either Palau or PNG, as well as a branch split to comprise shrimp from either northern Australia or Fiji. Clades 4 and 5 comprised shrimp only from Thailand and Western Australia, respectively, whereas Clade 6 included a branch comprising shrimp from Sri Lanka and a branch split to comprise shrimp from either Madagascar or India.

**Table 1:** Pair-wise comparisons of genetic distance (F_ST_ and Ø_ST_) among all 17 *Penaeus monodon* populations

	Mad	Ind	Sri	Bru	Thai	VCM	VCT	VBL	VBT	Phil	Taiw	WAU	NAU	EAU	Fiji	Pal	PNG
Mad	–	0.460	0.359	0.576	0.405	0.442	0.548	0.857	0.429	0.806	0.764		0.819	0.803	0.898	0.615	0.801
Ind	0.065	–	0.040	0.474	0.238	0.182	0.423	0.806	0.000	0.758	0.715		0.781	0.762	0.870	0.539	0.757
Sri	0.071	0.001	–	0.230	0.063	0.062	0.277	0.589	0.000	0.555	0.527		0.626	0.599	0.769	0.340	0.574
Bru	0.137	0.070	0.049	–	0.032	0.248	0.498	0.210	0.325	0.160	0.131		0.276	0.274	0.476	0.000	0.232
Thai	0.081	0.021	0.010	0.038	–	0.099	0.310	0.305	0.119	0.272	0.248		0.347	0.331	0.493	0.103	0.299
VCM	0.158	0.106	0.083	0.109	0.077	–	0.396	0.608	0.088	0.554	0.525		0.623	0.585	0.762	0.341	0.562
VCT	0.128	0.066	0.043	0.063	0.038	0.118	–	0.857	0.354	0.782	0.724		0.792	0.771	0.882	0.544	0.769
VBL	0.113	0.068	0.046	0.074	0.037	0.111	0.073	–	0.721	0.155	0.139		0.288	0.397	0.502	0.111	0.378
VBT	0.136	0.079	0.061	0.092	0.050	0.096	0.083	0.082	–	0.666	0.623		0.713	0.688	0.832	0.421	0.672
Phil	0.109	0.055	0.033	0.051	0.015	0.094	0.040	0.048	0.053	–	0.000		0.141	0.145	0.403	0.051	0.105
Taiw	0.113	0.050	0.033	0.057	0.017	0.084	0.056	0.039	0.055	0.016	–		0.068	0.076	0.341	0.033	0.037
WAU	0.109	0.048	0.039	0.072	0.028	0.102	0.082	0.078	0.078	0.041	0.043	–					
NAU	0.163	0.105	0.090	0.118	0.067	0.178	0.098	0.112	0.144	0.091	0.085	0.116	–	0.155	0.439	0.153	0.105
EAU	0.104	0.050	0.030	0.050	0.011	0.076	0.050	0.034	0.045	0.008	0.009	0.032	0.091	–	0.448	0.153	0.000
Fiji	0.154	0.107	0.082	0.104	0.056	0.116	0.090	0.104	0.108	0.052	0.056	0.094	0.101	0.051	–	0.311	0.418
Pal	0.096	0.034	0.020	0.039	0.003	0.071	0.042	0.041	0.055	0.017	0.018	0.025	0.067	0.009	0.054	–	0.117
PNG	0.111	0.047	0.031	0.044	0.006	0.097	0.052	0.035	0.068	0.020	0.012	0.048	0.071	0.005	0.055	0.004	–

F_ST_ values are shown below the diagonal and Ø_ST_ values are shown above the diagonal. F_ST_ values for the mtDNA-CR sequence of a single Western Australian shrimp are not shown.

Mad, Madagascar; Ind, India; Sri, Sri Lanka; Bru, Brunei; Thai, Thailand; VCM, Vietnam-CM; VCT, Vietnam-CT; VBL, Vietnam-BL; VBT, Vietnam-BT; Phil, Philippines; Taiw, Taiwan; WAU, Western Australia; NAU, northern Australia; EAU, eastern Australia; Pal, Palau; PNG, Papua New Guinea.

Not significant (dark shading), significant at *P* < 0.05 (light shading), significant at *P* < 0.01 (no shading).

**Figure 4:**
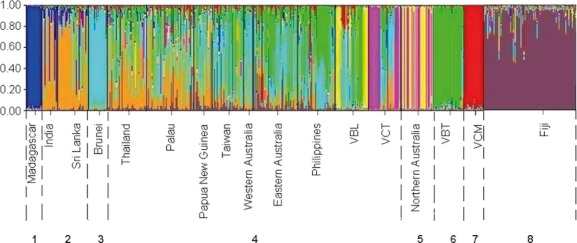
Bayesian STRUCTURE analysis of microsatellite genotypes clustered *Penaeus monodon* from the 17 Indo-Pacific localities into eight groups. Each vertical line pertains to an individual and the proportion of each color per individual represents its genetic composition from each the eight clusters (K = 8) inferred. Madagascar (1); India and Sri Lanka (2); Brunei (3); Thailand, Palau, PNG, Taiwan, Western Australia, eastern Australia, Philippines, Vietnam-BL, Vietnam-CT (4); North Australia (5); Vietnam-BT (6); Vietnam-CM (7); Fiji (8).

Bayesian analysis of microsatellite allele frequencies segregated the 615 *P. monodon* into eight population clusters (Fig. 4). These clusters comprised shrimp from Madagascar (Cluster #1), India and Sri Lanka (#2), Brunei (#3), Thailand, Palau, PNG, Taiwan, Western Australia, eastern Australia, the Philippines, Vietnam-BL, Vietnam-CT (#4), northern Australia (#5), Vietnam-BT (#6), Vietnam-CM (#7), and Fiji (#8). When the Bayesian analysis was repeated using the data derived for groups of 16 shrimp selected at random from each of the 17 populations, except for the Western Australian group that comprised only nine individuals, these groups segregated similarly into eight clusters comprising representatives of the same geographic cohorts (data not shown).

### Genetic diversity deduced from mtDNA-CR sequences

A total of 247 haplotypes were identified in alignments of a 418 nt mtDNA-CR sequence amplified from 369 *P. monodon* comprising representatives of each of the 17 geographic populations (Table 2). Of these haplotypes, 235 (95.1%) were unique. Unique haplotypes occurred among shrimp sourced from Madagascar, Brunei, Vietnam-CM, Vietnam-CT, Vietnam-BT, Western Australian, northern Australia, eastern Australia, and Fiji. Each of the remaining 12 haplotypes was shared between shrimp sourced from no more than two locations. Thai shrimp had the highest number of haplotypes shared with shrimp from elsewhere, with eight different haplotypes shared with shrimp from India (1), Sri Lanka (2), Taiwan (2), PNG (1), or Palau (2). The four remaining haplotypes were shared between shrimp from Vietnam-BL–Taiwan, Vietnam-BL–Palau, Philippines–Taiwan, and Taiwan–PNG. Haplotype diversity (h) was high among shrimp from most geographic locations, approaching 1.0 for shrimp from Madagascar, India, eastern Australia, and PNG. Among shrimp from northern Australia and from three of the four provinces in Vietnam, however, diversity was substantially lower (*h* < 0.7) (Table 2).

**Table 2:** Molecular diversity indices determined for mtDNA-CR sequences across 16 of the 17 *Penaeus monodon* populations

Location	*n*	N_*h*_	*h*	π	Fu's Fs	*P*-value	Tajima's D	*P*-value	*t* (years)
Mad	12	12	1.00	0.04	−3.45	0.04	−1.52	0.06	104,004
Ind	16	16	1.00	0.04	−5.45	0.01	−1.61	0.04	48,235
Sri	16	14	0.98	0.07	−6.22	0.42	0.64	0.80	
Bru	14	4	0.69	0.07	16.74	1.00	1.67	0.98	
Thai	40	35	0.99	0.08	−5.51	0.06	1.34	0.94	
VCM	15	2	0.48	0.07	26.00	1.00	2.38	0.99	
VCT	9	2	0.22	0.03	12.58	1.00	−2.03	0.00	
VBL	10	4	0.64	0.01	1.94	0.84	−0.46	0.35	
VBT	11	6	0.85	0.06	6.18	0.99	0.11	0.57	
Phil	14	11	0.96	0.03	−0.85	0.32	−0.12	0.48	
Taiw	23	18	0.97	0.04	−2.17	0.19	−1.04	0.15	
NAU	31	3	0.54	0.03	19.33	0.99	3.57	1.00	
EAU	25	25	1.00	0.03	−14.59	0.00	−0.59	0.31	164,705
Pal	33	29	0.99	0.07	−5.38	0.04	−0.03	0.58	19,527
PNG	18	18	1.00	0.03	−8.26	0.00	0.10	0.59	63,565
Fiji	81	48	0.98	0.01	−25.28	0.00	−1.51	0.04	60,470
Mean	23	15	0.83	0.04					

*n*, number of sequences; N_*h*_, number of haplotypes; *h*, haplotype diversity; π, nucleotide diversity; Fu's Fs and Tajima's D neutrality tests with significance *P*-values and *t*, time since population expansion. Data for Western Australia not included due to mtDNA-CR data being available for a single shrimp. Location codes are as described in Table 1.

**Figure 5:**
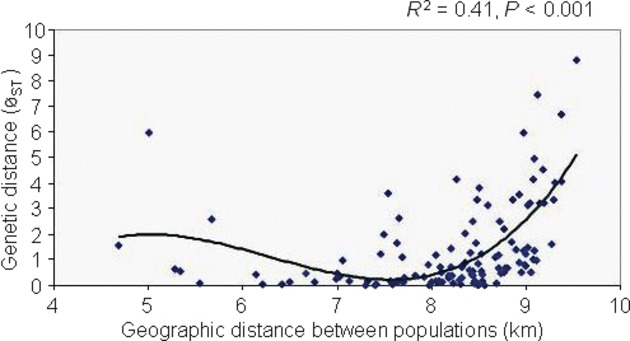
Mantel test (1000 permutations; isolation-by-distance significance *P* < 0.0001) correlations between genetic distance and geographical distance based on mtDNA-CR haplotype data on the 369 *Penaeus monodon* examined across the 17 Indo-Pacific localities.

In contrast to haplotype diversity, nucleotide sequence diversity (π) was low in the 418 nt mtDNA-CR sequence examined, ranging from 1% for shrimp from either Vietnam-BL or Fiji to 8% for shrimp from Thailand. Analysis of Molecular Variance analyses of sequence differences were used to calculate genetic distances (Ø_ST_) between the 17 shrimp populations, and identified that most were distinct (*P* < 0.05) (Table 1). The Mantel test deduced that genetic distances and the geographical distances between populations were correlated positively and followed a polynomial relationship (Fig. 5). However, Ø_ST_ values did not differ significantly (*P* > 0.05) in pair-wise comparisons of shrimp from India and either Sri Lanka or Vietnam-BT, Sri Lanka and either Thailand, Vietnam-CM or Vietnam-BT, Brunei and either Thailand or Palau, Vietnam-CM and Vietnam-BT, the Philippines and either Taiwan or Palau, and from Taiwan and either Palau or PNG (Table 1). These population relationships defined by mtDNA-CR sequence divergence did not, however, mirror relationships defined by F_ST_ values derived from eight microsatellite loci examined (Table 1).

### Population structure and expansion based on mtDNA-CR sequences

**Figure 6:**
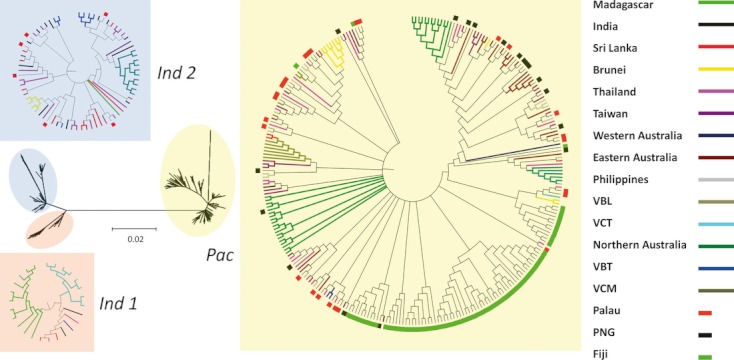
An unrooted Neighbor-Joining phylogenetic tree of 247 mtDNA-CR haplotypes identified among the 369 *Penaeus monodon* examined across the 17 Indo-Pacific localities showing their segregation into primary Pacific Ocean (*Pac*) and Indian Ocean groups (*Ind 1* and *Ind 2*). Within these groups, branches corresponding to partitions reproduced in <95% bootstrap replicates were collapsed. The origin of each individual within each branch is shown in the shaded circular diagrams by the use of location-specific line colors. Red, black and light-green colored bars on the outer rim of the *Pac* and *Ind 2* clade diagrams were used to highlight shrimp from the South Pacific locations of Palau, PNG and Fiji, respectively.

A Neighbor-Joining phylogenetic tree constructed from 247 *P. Monodon* mtDNA-CR haplotypes splits them into three primary grouping comprising shrimp predominantly from either Pacific Ocean (*Pac*) or Indian Ocean (*Ind 1* and *Ind 2*) origins (Fig. 6). The Pacific Ocean (*Pac*) group formed one large discrete clade (*n* = 273) including shrimp from Sri Lanka (*n =* 4), India (*n =* 1), Brunei (*n =* 10), Thailand (*n =* 21), Vietnam-CM (*n =* 5), Vietnam-CT (*n =* 1), Vietnam-BL (*n =* 10), Vietnam-BT (*n =* 3), the Philippines (*n = 14*), Taiwan (*n =* 22), northern Australia (*n =* 31), Western Australia (*n =* 1), eastern Australia (*n =* 25), PNG (*n =* 18), Palau (*n =* 27), and Fiji (*n =* 81) (Fig. 6).

The Indian Ocean group segregated into two discrete clades (*Ind 1* and *Ind 2*). *Ind 1* clade comprised 26 shrimp including Vietnam-CT (*n* = 8), India (*n* = 2), Sri Lanka (*n* = 2), Thailand (*n* = 2), Vietnam-BT (*n* = 1), and Madagascar (*n* = 11). *Ind 2* clade comprised 70 shrimp including Vietnam-BT (*n* = 8), India (*n* = 13), Sri Lanka (*n* = 10), Thailand (*n* = 17), Palau (*n* = 6), Brunei (*n* = 4), Vietnam-CM (*n* = 10), Taiwan (*n* = 1), and Madagascar (*n* = 1) (Fig. 6).

Examining the mtDNA-CR haplotype relationships showed them broadly to conform to one of three types in which (1) 80–90% of shrimp from a location clustered together (i.e., Madagascar, India, Vietnam-CT and Vietnam-BL, Western Australia, and Fiji), (2) 50–79% of shrimp from a location cluster together (i.e., Brunei, Vietnam-CM and Vietnam-BT, Philippines, Taiwan, Sri Lanka, northern Australia, eastern Australia, Palau, and PNG) or (3) <50% of shrimp from a location clustered together (i.e., Thailand).

Using Fu's Fs test for neutrality (i.e., mutation-drift equilibrium), analysis of mtDNA-CR sequences across the 17 geographic groups identified that six groups (i.e., Madagascar, India, eastern Australia, Palau, PNG, and Fiji) generated significant (*P* < 0.05) negative Fs values suggestive of previous population expansion events (Table 2). Analysis of the same sequences using a Tajima D test for neutrality only identified significant (*P* < 0.05) negative D values for shrimp from India, Vietnam-CT, and Fiji (Table 2). To look for further evidence supporting population expansion within each of the six geographic groups that showed negative Fs values, mtDNA-CR sequences were compared by mismatch analysis to identify unimodal distributions of nucleotide mismatches (data not shown). However, such distribution was only identified with the shrimp from Fiji. From τ values generated from the mismatch analysis, estimated time of past population expansions for the six groups with negative Fs values were calculated using the equation T = τ/2u and to have occurred between 20,000 and 165,000 years ago (Table 2).

## Discussion

### Genetic diversity and population structure of *P. monodon* in South Pacific in relation to the rest of Indo-Pacific *P. monodon* populations

Here, we have examined genetic relatedness of 615 *P. monodon* sourced from 17 localities spread widely across its Indo-Pacific distribution range, and including the Pacific Island countries of Palau, PNG, and Fiji. The overall levels of genetic diversity of *P. monodon* in Palau, PNG, and Fiji were very high. These were clearly demonstrated by the percentage of heterozygosity (0.82–0.91), allelic richness (6.85–9.69), and mean number of alleles per locus (13.5–19.8) of microsatellites.

The examination of the overall genetic structure of *P. monodon* from its Indo-Pacific distribution range using microsatellite markers identified substantially more genetic structures than previous studies for this species (You et al. 2008). The Bayesian STRUCTURE analysis of genotypes segregated *P. monodon* from 17 geographic locations into eight clusters comprised of shrimp from Madagascar (1), India and Sri Lanka (2), Thailand, Palau, PNG, Taiwan, Western Australia, eastern Australia, Philippines, Vietnam-BL and Vietnam-CT (3), northern Australia (4), Vietnam-BT (5), Brunei (6), Vietnam-CM (7), and Fiji (8). The results were well supported by an isolation-by-distance organization (r^2^ = 0.41, *P* < 0.001). A recent similar study of genetic diversity of *P. monodon* from Australia, Kenya, Madagascar, the Philippines, Taiwan, Thailand, and Vietnam delineated distinct Pacific Ocean, Indian Ocean, and West Thailand clusters (You et al. 2008). This discrepancy likely stems from the *P. monodon* examined here being sourced from a wider geographical range including Brunei, Fiji, India, Sri Lanka, Palau, and PNG and possibly by the microsatellites examined comprising tri- and tetranucleotide repeats that are known to display higher levels of allelic polymorphism (Edwards et al. 1991; Zheng et al. 2010).

In comparison to genetic structure of *P. Monodon* from the rest of Indo-Pacific distribution range, *P. monodon* in South Pacific were clustered into Pacific Ocean phylogenetic group. Interestingly, both mtDNA-CR hyplotype and microsatellite heterozogisity analyses demonstrate that the shrimp from Fiji were unique and genetically distinct from the rest of the Pacific group. The shrimp from Palau and PNG were genetically more similar to Southeast Asian and East-Australian shrimp strains, respectively.

### Ancestral dispersal of *P. monodon* across the Indo-Pacific region

Based on mtDNA RFLP analyses, (Benzie et al. (2002) hypothesized that ancestrally, *P. monodon* might have dispersed easterly from Africa to occupy their current geographic range. However, the population structures and diversities identified here from analyses of microsatellite loci genotypes and mtDNA-CR haplotypes suggest a more complex pattern of dispersal. Indeed, based on the genetic distinctions between disparate populations across its Indo-Pacific distribution range, it is tempting to hypothesize that a progenitor *P. monodon* species might have had ancestral origins restricted to tropical and subtropical coastlines of the easterly region of the Gondwana supercontinent. With this possibility, on Gondwana fragmenting, shrimp resident to newly formed coastlines of East Africa, Madagascar, the India subcontinent and Australia with favorable habitats and water temperatures could have dispersed *P. monodon* through continual drift (Condie 2005). Subsequent ice age events that resulted in sea level lowering may have provided subsequent opportunities for further dispersal across its current natural distribution range.

Microsatellite allele types and mtDNA-CR haplotypes detected among *P. monodon* from Madagascar distinguished them from shrimp from elsewhere. Due to the geographic remoteness of Madagascar from the nearest easterly study locations (Sri Lanka and India), such genetic distinction was expected. This could also explain with the Member–Vagrant evolutionary model that restricted gene flows result in locally adapted gene pools and stable genetic structures.

Conversely for reasons of geographic proximity, it was not surprising that *P. monodon* from Sri Lanka and India could not be distinguished by either analysis method. The same was found with most shrimp examined from the several South-East Asian study locations, suggesting a common ancestry and higher levels of recent gene flow perpetuated by natural intermixing of proximal populations or intermixing due to shrimp translocations for aquaculture purposes. These fit well with a metapopulation evolutionary model, that is, highly frequent gene flows reduce genetic diversity between local subpopulations causing recurring extinction–recolonization events (Hanski 1998; Ensing et al. 2011).

Within the region, however, shrimp from Thailand possessed more varied mtDNA-CR haplotypes, many of which were common to shrimp from Indian Ocean and Pacific Ocean locations. This study group comprised *P. monodon* originating from both the Gulf of Thailand and the Andaman Sea on either side of the Thai-Malay Peninsula, which might in part explain these broader relationships. However, the aquaculture of *P. monodon* in Thailand for over three decades, and the common intra-country trade in broodstock to meet Thai hatchery needs, is also likely to have contributed.

With shrimp originating from the four proximal locations in southern Vietnam, those from CM and BT provinces segregated into distinct clusters whereas those from BL and CT provinces clustered together among shrimp from other South-East Asian locations. However, as these samples were postlarvae collected from hatcheries, and as the source or number of wild broodstock from which they were derived is not known, rather than indicating differences in gene flow between these provinces, the data likely reflect more or less localized origins of broodstock sourced by hatcheries. Moreover, the generally small sample sizes, the lack of any consistent congruence between microsatellite and mtDNA-CR data, and the knowledge that many South-East Asian countries have long histories of trading in live *P. monodon* broodstock for aquaculture use make it difficult to draw definitive conclusions on the ancestral origins and genetic relationships of the shrimp examined from this region.

Interestingly, microsatellite genotypes identified among *P. monodon* from Western Australia, the easterly boundary of the Indian Ocean, clustered them among shrimp from Pacific Ocean locations, as did the single mtDNA-CR haplotype identified. These data are consistent with mtDNA RFLP data reported by Benzie et al. (2002), and with the upwelling of deep cold water along the North-Western coastline of Australia that has likely existed since the Late Miocene period precluding gene flow between shrimp populations separated by this natural barrier (Williams and Benzie 1997, 1998).

Both microsatellite genotyping and mtDNA-CR haplotype data segregated shrimp from northern Australia into two gene pools, one related to shrimp mostly from Southeast Asia and the other related to shrimp mostly from eastern Australia and PNG. Although the exact capture locations of the *P. monodon* are not known, these data suggest northern Australia has experienced recent gene flows from these regions possibly during the Pleistocene period of global glaciations and lowered sea levels (Torgersen et al. 1983, 1985).

Both analyses identified among *P. monodon* from eastern Australia consistently clustered them with PNG shrimp, reflecting high ancestral gene flows between these regions. Although likely mediated by past linking of coastlines during glaciations, the extremely low F_ST_ value (0.005) deduced from microsatellite analyses suggest that these populations might be mixing perpetually due to their proximity and the relative shallowness of Torres Strait and its many Islands providing opportunities for Island hopping.

### Population expansion of *P. Monodon* in Indo-Pacific region

Three demographic tests (Fu's Fs, Tajima's D and mismatch distribution) were performed to detect possible population expansion of *P. Monodon* from 17 locations using mtDNA-CR sequence. Although all three methods unanimously identified recent population expansion in the shrimp from Fiji, they also generated different results. For example Fu's Fs test predicted population expansions among *P. monodon* from Madagascar and India (Indian Ocean) as well as eastern Australia, PNG, Palau and more so from Fiji (Pacific Ocean). However, Tajima's D neutrality test only provided partial support to the Fu's Fs findings on India and Fiji, and mismatch distribution confirmed the results on Fiji only. Small population sizes may have contributed to the discrepancy of the results from different methods. On the other hand, the results were not unexpected. The study by Ramos-Onsins and Rozas (2002) found that out of 17 statistical methods applied for estimating population expansion (including three methods used here), Fu's Fs test was the most powerful test and the mismatch distribution was the most conservative method.

The population expansions predicted from mismatch distributions were dated between 20,000 and 165,000 years ago, thus traversing the Pleistocene ice age period. Such time frames for population expansion generally agree with data on numerous Indo-Pacific distributed species that now display a wide variety of population structures, yet all share the impact of Pliocene/Pleistocene glacial cycles (Imron et al. 2007; Dubey et al. 2010; Pavlova et al. 2010; Ravago-Gotanco and Juinio-Menez 2010; Winters et al. 2010 and Gaither et al. 2011).

Based on mtDNA RFLP data, Benzie et al. (2002) hypothesized that land bridges and common coastlines that formed during repeated periods of global cooling and lowered sea levels allowed *P. monodon* to influx into new areas, with subsequent population isolation and remixing events occurring in localized regions. Although consistent with this scenario, data reported here expand this to suggest that, in addition to the populations currently indigenous to eastern Africa and eastern Australia, population expansion also occurred into the Oceanic region, the easterly extreme of the *P. monodon* distribution range. In addition, by investigating shrimp from more locations, data pointed to *P. monodon* from eastern Australia experiencing the earliest expansion followed by eastern Africa, as based on data on shrimp from Madagascar, with a more recent series of expansions occurring in India, PNG/Palau and Fiji. A mismatch analysis of mtDNA-CR haplotypes to examine for demographic expansions only generated a smooth unimodal curve with the shrimp originating from Fiji, suggesting it has experienced a relatively more recent influx of new haplotypes.

It is worthwhile to mention that these neutrality tests certainly provide us insights whether or not population expansion (or growth) has been significant or extensive, but they are unable to accurately estimate the direction and shape of population expansion through time. Therefore, additional analysis using Bayesian Skyline Plot would be needed to provide more information on estimated effective population sizes over time.

### *P. monodon* colonization of South Pacific islands

The islands of the South Pacific are generally not continental in origin. Palau and Fiji were formed by complex geological processes of plate subduction and uplift of volcanic intrusions (Neall and Trewick 2008). From genetic analyses of *P. monodon* from the three South Pacific islands examined, their distinct genetic makeup was indicative of them having established more recently, most likely from small numbers of founder shrimp which has restricted gene pool sizes at any particular location. Together with the Malay Peninsula, the Philippines and Indonesia, PNG comprises part of the Malesia biogeographical region which shares tropical flora originating mainly from nearby Asia (Donaldson 2002; Keppel et al. 2009; van Balgooy 1971). The main archipelago of Palau also boarders this region and is only ∼560 km east of the Philippines. There are geological, biological and genetic data on modern communities of biota traversing South-East Asia and Island Nations further into the Pacific that define the processes by which long-distance dispersal (LDD) has occurred (Keppel et al. 2009). The genetic similarities between *P. monodon* from South-East Asian localities and PNG/Palau suggest that this species has been provided similar opportunities for dispersal, most likely when land masses and thus coastlines merged in times of lowered sea levels.

Heterozygosity in both microsatellite loci and mtDNA-CR haplotypes clearly demarcated Fiji shrimp as genetically unique compared to other populations, suggesting their derivation from a relatively small founder population that has remained isolated due to its geographic remoteness. Such genetic separation due to the geographic isolation of Fiji has also been found recently among non-migratory reef fish species examined from Indo-West Pacific locations including Indonesia, PNG, the Solomon Islands and Fiji (Drew et al. 2010). The unique makeup Fiji shrimp identified here is supported by mtDNA RFLP data on *P. monodon* from Fiji as well as *Penaeus japonicus* from Malaysia and Australia from which it was hypothesized that, after colonization, *P. monodon* now indigenous to the Melanesian region of the Oceanic Islands might have become isolated (Bouchon et al. 1994). Despite their uniqueness, some Fiji shrimp showed low-level genetic similarities to shrimp from the Philippines. The likely explanation for these similarities is Fiji importing *P. monodon* from the Philippines in 1975 as part of a United Nations Development Program to assess their aquaculture potential (Choy 1981), and the subsequent release of progeny of these broodstock into the wild to interbreed with indigenous shrimp.

Some *P. monodon* from eastern Australia and some from PNG possessed mtDNA-CR haplotypes that grouped them within the predominant narrow genetic clade of shrimp from Fiji, thus also supporting the potential for easterly LDD to South Pacific islands, with populations becoming established from small numbers of founder shrimp. Data on *P. monodon* from Fiji indicate that largely they still remain a distinct genetic population, however, most likely owing to their geographic isolation. Voloch et al. (2009) estimated that Penaeid shrimp dispersal might have occurred widely 20–43 million years ago, which coincides with the estimated ages of many Oceanic islands since emerging from the sea (Gill 1970; Bain 1973). Thus, it may be valuable to identify the genetic makeup of *P. monodon* from other remote locations distributed across the Melanesian and South Pacific regions such as the Solomon Islands, Vanuatu, new Caledonia, Samoa, Tonga, the Cook Islands and French Polynesia to determine whether or not LDD has similarly been involved in their colonization by this shrimp species.

## Conclusion

By examining heterozygosity among eight microsatellite loci and mtDNA-CR sequence differences among 17 populations of *P. monodon* sampled across its Indo-Pacific distribution range from Madagascar to Fiji, data supported shrimp from the South Pacific (Palau, PNG and Fiji) being colonized relatively recently by neighboring populations closest in proximity. The findings clearly demonstrate that *P. monodon* distributed across the Indo-Pacific region have evolved following both Member–Vagrant and Metapopulation evolutionary models. The data are also consistent with the current dispersal patterns and population structures of *P. monodon* being mediated over long geological time frames most likely by global glaciation periods linking coastlines now well separated and possibly by ancient progenitor populations being dispersed by continental drift of northeasterly fragments of the Gondwana supercontinent.

## Supporting Information

**Table S1.** Within-population genetic diversity per microsatellite loci across all 17 *Penaeus monodon* populations.
