# Supplementary material for: Genetic analysis of Black Tiger shrimp (Penaeus monodon) across its natural distribution range reveals more recent colonization of Fiji and other South Pacific islands
Source: Ecol Evol. 2012 Jul 22;2(8):2057–71. doi: 10.1002/ece3.316 (PMC3434007; doi:10.1002/ece3.316)
Supplement: Supplementary file 1 [file ece30002-2057-SD1.doc]

| **Supplementary Table 1 Within-population genetic diversity per microsatellite loci across all 17 *P. monodon* populations** | | | | | | | | | | | |  |
| --- | --- | --- | --- | --- | --- | --- | --- | --- | --- | --- | --- | --- |
| n, Number of individuals genotyped; A, Number of alleles; Ap, Number of private alleles; Ar, Allelic richness; He, Expected heterozygosity | | | | | | | | | | | |  |
| Ho, Observed heterozygosity; Fis, Within-population genetic diversity | | | | | | | | | | | |  |
|  | | | | | | | | | | | |  |
|  | **Pm8** | **Pm2D2** | **Pm14** | **Pm4C** | **DTLPm313** | **Pm9** | **Pm9GG** | **Pm16** | **Pm8A2** | **Pm6** | **Pm11AH** | **Mean** |
| **Allele range (bp)** | 120-160 | 210-390 | 151-213 | 183-272 | 521-600 | 140-287 | 215-270 | 100-235 | 245-340 | 230-350 | 115-220 |  |
|  |  |  |  |  |  |  |  |  |  |  |  |  |
| **MAD (16)** |  |  |  |  |  |  |  |  |  |  |  |  |
| n | 16 | 15 | 0 | 10 | 16 | 16 | 16 | 16 | 15 | 0 | 16 |  |
| A | 5 | 15 | 0 | 6 | 11 | 5 | 6 | 13 | 8 | 0 | 13 | 7.5 |
| Ap | 10 | 8 | 0 | 0 | 1 | 1 | 0 | 1 | 0 | 0 | 0 | 1.9 |
| Ar | 4.49 | 11.70 |  | 5.80 |  | 4.79 | 5.78 | 9.75 | 7.33 |  | 9.54 | 7.40 |
| He | 0.734 | 0.947 |  | 0.826 | 0.915 | 0.784 | 0.839 | 0.867 | 0.862 |  | 0.905 | 0.853 |
| Ho | 0.625 | 0.600 |  | 0.600 | 0.938 | 0.625 | 0.875 | 0.938 | 0.800 |  | 0.625 | 0.736 |
| Fis | 0.153 | **0.375** |  | 0.285 | -0.025 | 0.208 | -0.045 | -0.084 | 0.074 |  | **0.317** | 0.140 |
| **IND (17)** |  |  |  |  |  |  |  |  |  |  |  |  |
| n | 17 | 17 | 15 | 17 | 12 | 13 | 16 | 17 | 17 | 17 | 17 |  |
| A | 9 | 22 | 14 | 18 | 10 | 12 | 11 | 22 | 15 | 12 | 17 | 14.7 |
| Ap | 0 | 4 | 2 | 1 | 0 | 2 | 0 | 3 | 1 | 0 | 5 | 1.6 |
| Ar | 7.10 | 13.61 |  | 12.41 |  | 10.33 | 9.47 | 14.46 | 10.82 |  | 12.21 | 11.30 |
| He | 0.774 | 0.970 | 0.913 | 0.948 | 0.902 | 0.92 | 0.923 | 0.975 | 0.929 | 0.927 | 0.945 | 0.921 |
| Ho | 0.824 | 0.706 | 0.667 | 0.941 | .0001 | 0.667 | 0.813 | 0.647 | 0.765 | 0.765 | 0.882 | 0.789 |
| Fis | -0.067 | **0.278** | **0.276** | 0.008 | -0.114 | **0.285** | 0.124 | **0.343** | **0.181** | **0.179** | 0.068 | 0.142 |
| **SRI (32)** |  |  |  |  |  |  |  |  |  |  |  |  |
| n | 32 | 32 | 32 | 32 | 32 | 32 | 32 | 32 | 32 | 32 | 32 |  |
| A | 10 | 30 | 12 | 23 | 15 | 13 | 15 | 30 | 19 | 23 | 31 | 20.1 |
| Ap | 0 | 7 | 0 | 3 | 2 | 2 | 1 | 4 | 1 | 0 | 3 | 2.1 |
| Ar | 7.33 | 14.01 |  | 12.29 |  | 8.09 | 9.80 | 13.64 | 10.87 |  | 14.46 | 11.31 |
| He | 0.843 | 0.968 | 0.795 | 0.95 | 0.916 | 0.866 | 0.915 | 0.964 | 0.927 | 0.952 | 0.973 | 0.915 |
| Ho | 1 | 0.875 | 0.563 | 0.875 | 0.906 | 0.688 | 0.938 | 0.781 | 0.875 | 0.906 | 0.969 | 0.852 |
| Fis | -0.19 | **0.098** | **0.296** | **0.081** | **0.011** | **0.209** | -0.025 | **0.192** | 0.057 | 0.049 | 0.005 | 0.071 |
| **BRU (20)** |  |  |  |  |  |  |  |  |  |  |  |  |
| n | 20 | 19 | 18 | 20 | 0 | 20 | 20 | 19 | 20 | 20 | 20 |  |
| A | 7 | 11 | 7 | 13 | 0 | 6 | 9 | 14 | 9 | 9 | 11 | 8.7 |
| Ap | 0 | 0 | 0 | 0 | 0 | 0 | 1 | 1 | 0 | 0 | 2 | 0.4 |
| Ar | 6.02 | 8.67 |  | 9.39 |  | 4.45 | 7.46 | 10.40 | 7.53 |  | 8.39 | 7.79 |
| He | 0.797 | 0.892 | 0.614 | 0.905 |  | 0.613 | 0.85 | 0.927 | 0.869 | 0.871 | 0.892 | 0.823 |
| Ho | 0.7 | 0.842 | 0.389 | 1 |  | 0.5 | 0.95 | 1 | 0.8 | 0.55 | 0.85 | 0.758 |
| Fis | 0.125 | 0.057 | **0.374** | -0.108 |  | 0.188 | -0.121 | -0.081 | 0.082 | **0.374** | 0.049 | 0.094 |
| **THAI (42)** |  |  |  |  |  |  |  |  |  |  |  |  |
| n | 42 | 42 | 41 | 42 | 36 | 39 | 42 | 42 | 42 | 41 | 37 |  |
| A | 13 | 32 | 14 | 17 | 16 | 9 | 16 | 26 | 21 | 24 | 29 | 19.7 |
| Ap | 1 | 3 | 0 | 1 | 11 | 1 | 0 | 2 | 3 | 1 | 3 | 2.4 |
| Ar | 8.39 | 13.05 |  | 10.11 |  | 6.69 | 9.84 | 12.41 | 10.61 |  | 11.80 | 10.36 |
| He | 0.877 | 0.954 | 0.898 | 0.908 | 0.919 | 0.844 | 0.917 | 0.95 | 0.923 | 0.951 | 0.932 | 0.916 |
| Ho | 0.881 | 0.857 | 0.425 | 0.833 | 0.625 | 0.744 | 0.976 | 0.881 | 0.881 | 0.707 | 0.929 | 0.794 |
| Fis | -0.005 | **0.103** | **0.53** | 0.084 | **0.323** | **0.121** | -0.066 | 0.073 | 0.046 | 0.258 | 0.004 | 0.134 |
| **VIETCM (22)** |  |  |  |  |  |  |  |  |  |  |  |  |
| n | 21 | 22 | 22 | 22 | 22 | 21 | 22 | 22 | 22 | 21 | 20 |  |
| A | 5 | 6 | 6 | 7 | 4 | 4 | 7 | 7 | 7 | 8 | 6 | 6.1 |
| Ap | 0 | 0 | 0 | 0 | 0 | 0 | 1 | 0 | 0 | 0 | 0 | 0.1 |
| Ar | 4.34 | 5.33 |  | 6.31 |  | 3.97 | 6.15 | 5.56 | 5.40 |  | 4.94 | 5.25 |
| He | 0.647 | 0.771 | 0.704 | 0.84 | 0.574 | 0.738 | 0.837 | 0.796 | 0.785 | 0.852 | 0.71 | 0.750 |
| Ho | 0.762 | 1 | 0.909 | 0.955 | 0.45 | 0.857 | 0.955 | 0.818 | 1 | 0.952 | 0.727 | 0.853 |
| Fis | -0.183 | -0.307 | -0.3 | -0.14 | 0.221 | -0.167 | -0.144 | -0.029 | -0.282 | -0.12 | -0.024 | -0.134 |
| **VIETCT (33)** |  |  |  |  |  |  |  |  |  |  |  |  |
| n | 33 | 32 | 33 | 33 | 32 | 33 | 33 | 33 | 33 | 32 | 33 |  |
| A | 7 | 15 | 9 | 12 | 12 | 6 | 11 | 15 | 16 | 15 | 10 | 11.6 |
| Ap | 0 | 0 | 0 | 0 | 1 | 0 | 0 | 2 | 2 | 1 | 0 | 0.5 |
| Ar | 5.46 | 9.13 |  | 7.94 |  | 5.18 | 7.81 | 8.97 | 9.68 |  | 7.24 | 7.67 |
| He | 0.741 | 0.898 | 0.854 | 0.852 | 0.858 | 0.73 | 0.857 | 0.893 | 0.907 | 0.892 | 0.859 | 0.849 |
| Ho | 0.758 | 1 | 0.879 | 0.788 | 1 | 0.818 | 0.97 | 0.727 | 0.938 | 0.688 | 1 | 0.870 |
| Fis | -0.023 | -0.115 | -0.029 | **0.076** | -0.168 | -0.124 | -0.134 | **0.188** | -0.034 | **0.232** | -0.167 | -0.027 |
| **VIETBL (35)** |  |  |  |  |  |  |  |  |  |  |  |  |
| n | 35 | 35 | 34 | 35 | 34 | 35 | 35 | 35 | 35 | 35 | 35 |  |
| A | 7 | 14 | 8 | 8 | 10 | 5 | 16 | 10 | 9 | 15 | 9 | 10.1 |
| Ap | 0 | 1 | 0 | 0 | 0 | 0 | 1 | 1 | 0 | 0 | 0 | 0.3 |
| Ar | 5.69 | 10.27 |  | 7.19 |  | 4.66 | 9.48 | 7.25 | 6.84 |  | 6.72 | 7.26 |
| He | 0.769 | 0.928 | 0.852 | 0.869 | 0.892 | 0.767 | 0.891 | 0.848 | 0.853 | 0.917 | 0.836 | 0.857 |
| Ho | 0.8 | 0.943 | 0.206 | 0.914 | 0.971 | 0.714 | 0.971 | 0.857 | 0.943 | 0.857 | 0.857 | 0.821 |
| Fis | -0.04 | -0.016 | **0.761** | -0.053 | -0.089 | 0.07 | -0.092 | -0.011 | -0.107 | **0.066** | -0.026 | 0.042 |
| **VIETBT (35)** |  |  |  |  |  |  |  |  |  |  |  |  |
| n | 35 | 35 | 34 | 35 | 34 | 35 | 35 | 35 | 35 | 35 | 35 |  |
| A | 6 | 16 | 8 | 12 | 12 | 8 | 12 | 11 | 12 | 16 | 12 | 11.4 |
| Ap | 0 | 2 | 0 | 0 | 0 | 0 | 1 | 0 | 0 | 0 | 0 | 0.3 |
| Ar | 4.30 | 9.44 |  | 8.11 |  | 6.07 | 7.64 | 6.83 | 7.12 |  | 7.77 | 7.16 |
| He | 0.649 | 0.901 | 0.821 | 0.886 | 0.858 | 0.831 | 0.865 | 0.783 | 0.798 | 0.902 | 0.869 | 0.833 |
| Ho | 0.743 | 0.971 | 0.735 | 0.857 | 1 | 0.943 | 0.971 | 0.429 | 0.914 | 0.886 | 0.857 | 0.846 |
| Fis | -0.147 | -0.08 | 0.106 | 0.033 | -0.169 | -0.137 | -0.126 | **0.457** | -0.149 | 0.019 | 0.014 | -0.016 |
| **PHIL (39)** |  |  |  |  |  |  |  |  |  |  |  |  |
| n | 39 | 39 | 39 | 39 | 39 | 39 | 39 | 39 | 39 | 39 | 37 |  |
| A | 12 | 19 | 12 | 14 | 12 | 10 | 10 | 23 | 15 | 17 | 14 | 14.4 |
| Ap | 1 | 0 | 0 | 1 | 0 | 1 | 1 | 1 | 0 | 1 | 0 | 0.5 |
| Ar | 7.87 | 10.37 |  | 7.92 |  | 5.87 | 7.32 | 11.70 | 9.26 |  | 7.49 | 8.47 |
| He | 0.85 | 0.918 | 0.867 | 0.869 | 0.87 | 0.813 | 0.843 | 0.944 | 0.901 | 0.91 | 0.854 | 0.876 |
| Ho | 0.846 | 0.949 | 0.842 | 0.897 | 0.892 | 0.897 | 0.846 | 0.872 | 0.923 | 0.846 | 0.949 | 0.887 |
| Fis | 0.005 | -0.033 | 0.029 | -0.033 | -0.026 | -0.105 | -0.004 | **0.077** | -0.025 | **0.071** | -0.112 | -0.014 |
| **TAIW (33)** |  |  |  |  |  |  |  |  |  |  |  |  |
| n | 33 | 32 | 33 | 33 | 28 | 31 | 33 | 32 | 31 | 32 | 32 |  |
| A | 10 | 24 | 11 | 13 | 11 | 8 | 13 | 19 | 14 | 21 | 14 | 14.4 |
| Ap | 0 | 2 | 0 | 1 | 0 | 1 | 0 | 0 | 0 | 0 | 0 | 0.4 |
| Ar | 6.99 | 12.25 |  | 8.49 |  | 6.07 | 8.07 | 11.19 | 9.53 |  | 7.29 | 8.74 |
| He | 0.834 | 0.947 | 0.87 | 0.879 | 0.891 | 0.807 | 0.849 | 0.935 | 0.912 | 0.95 | 0.792 | 0.879 |
| Ho | 0.788 | 0.969 | 0.758 | 1 | 0.857 | 0.71 | 0.848 | 0.75 | 0.806 | 0.906 | 0.656 | 0.823 |
| Fis | 0.056 | -0.023 | 0.131 | -0.14 | 0.039 | 0.122 | **0.001** | **0.2** | **0.118** | 0.047 | 0.174 | 0.066 |
| **WAUST (9)** |  |  |  |  |  |  |  |  |  |  |  |  |
| n | 9 | 9 | 8 | 9 | 9 | 9 | 9 | 9 | 9 | 9 | 9 |  |
| A | 7 | 10 | 9 | 7 | 9 | 3 | 9 | 8 | 8 | 8 | 10 | 8.0 |
| Ap | 0 | 1 | 0 | 0 | 1 | 0 | 1 | 0 | 0 | 1 | 1 | 0.5 |
| Ar | 7.00 | 10.00 |  | 7.00 |  | 3.00 | 9.00 | 8.00 | 10.00 |  | 10.00 | 8.00 |
| He | 0.876 | 0.928 | 0.933 | 0.824 | 0.928 | 0.686 | 0.915 | 0.889 | 0.915 | 0.882 | 0.876 | 0.877 |
| Ho | 0.778 | 1 | 0.875 | 0.556 | 0.889 | 0.667 | 0.778 | 0.667 | 0.778 | 0.778 | 0.889 | 0.787 |
| Fis | 0.118 | -0.083 | 0.067 | **0.339** | 0.045 | 0.03 | 0.158 | **0.262** | 0.158 | 0.125 | -0.016 | 0.109 |
| **NAUST (40)** |  |  |  |  |  |  |  |  |  |  |  |  |
| n | 40 | 40 | 40 | 40 | 39 | 35 | 39 | 40 | 40 | 40 | 39 |  |
| A | 2 | 8 | 8 | 7 | 10 | 5 | 4 | 10 | 9 | 10 | 14 | 7.9 |
| Ap | 0 | 3 | 3 | 1 | 4 | 0 | 0 | 1 | 4 | 4 | 3 | 2.1 |
| Ar | 2.00 | 6.09 |  | 6.08 |  | 4.90 | 3.86 | 7.26 | 7.20 |  | 9.42 | 5.85 |
| He | 0.498 | 0.788 | 0.83 | 0.822 | 0.852 | 0.796 | 0.671 | 0.872 | 0.862 | 0.861 | 0.909 | 0.796 |
| Ho | 0.575 | 0.7 | 0.875 | 0.9 | 0.846 | 0.886 | 0.718 | 0.825 | 0.821 | 0.925 | 1 | 0.825 |
| Fis | -0.156 | 0.113 | -0.055 | -0.096 | 0.007 | -0.115 | -0.07 | 0.055 | 0.049 | -0.075 | -0.101 | -0.040 |
| **EAUST (46)** |  |  |  |  |  |  |  |  |  |  |  |  |
| n | 46 | 46 | 46 | 46 | 46 | 45 | 46 | 45 | 46 | 43 | 42 |  |
| A | 13 | 23 | 12 | 17 | 20 | 8 | 16 | 23 | 16 | 24 | 17 | 17.2 |
| Ap | 2 | 2 | 0 | 1 | 0 | 0 | 2 | 3 | 1 | 1 | 1 | 1.2 |
| Ar | 7.63 | 11.70 |  | 9.55 |  | 5.23 | 8.78 | 10.43 | 9.18 |  | 8.66 | 8.90 |
| He | 0.853 | 0.944 | 0.893 | 0.91 | 0.923 | 0.79 | 0.882 | 0.918 | 0.899 | 0.945 | 0.872 | 0.894 |
| Ho | 0.804 | 0.848 | 0.87 | 0.87 | 0.905 | 0.778 | 0.826 | 0.667 | 0.848 | 0.909 | 0.913 | 0.840 |
| Fis | 0.057 | **0.103** | 0.026 | 0.045 | 0.02 | 0.016 | **0.064** | **0.276** | 0.057 | 0.039 | -0.047 | 0.060 |
| **FIJI (125)** |  |  |  |  |  |  |  |  |  |  |  |  |
| n | 125 | 125 | 125 | 121 | 121 | 125 | 124 | 124 | 123 | 123 | 123 |  |
| A | 9 | 19 | 13 | 14 | 16 | 6 | 14 | 16 | 13 | 20 | 14 | 14.0 |
| Ap | 0 | 0 | 0 | 0 | 0 | 0 | 2 | 0 | 0 | 2 | 0 | 0.4 |
| Ar | 5.28 | 9.42 |  | 8.42 |  | 4.01 | 5.95 | 6.75 | 7.35 |  | 7.61 | 6.85 |
| He | 0.688 | 0.899 | 0.882 | 0.862 | 0.884 | 0.652 | 0.772 | 0.739 | 0.853 | 0.862 | 0.866 | 0.814 |
| Ho | 0.72 | 0.936 | 0.88 | 0.901 | 0.892 | 0.568 | 0.823 | 0.645 | 0.886 | 0.846 | 0.879 | 0.816 |
| Fis | -0.046 | -0.042 | 0.002 | -0.045 | **-0.008** | **0.13** | -0.066 | **0.128** | -0.039 | 0.019 | -0.016 | 0.002 |
| **PAL (50)** |  |  |  |  |  |  |  |  |  |  |  |  |
| n | 50 | 50 | 48 | 50 | 50 | 50 | 50 | 49 | 50 | 50 | 50 |  |
| A | 13 | 27 | 16 | 21 | 14 | 12 | 16 | 26 | 21 | 27 | 25 | 19.8 |
| Ap | 0 | 3 | 1 | 2 | 0 | 2 | 2 | 4 | 1 | 4 | 3 | 2.0 |
| Ar | 7.15 | 11.95 |  | 10.30 |  | 6.95 | 8.91 | 11.15 | 11.24 |  | 9.89 | 9.69 |
| He | 0.760 | 0.940 | 0.563 | 0.880 | 0.860 | 0.780 | 0.860 | 0.755 | 0.900 | 0.900 | 0.740 | 0.813 |
| Ho | 0.843 | 0.946 | 0.903 | 0.919 | 0.907 | 0.841 | 0.879 | 0.927 | 0.937 | 0.952 | 0.904 | 0.905 |
| Fis | 0.100 | **0.007** | **0.380** | **0.043** | 0.052 | 0.073 | 0.022 | **0.187** | 0.040 | **0.055** | **0.183** | 0.104 |
| **PNG (20)** |  |  |  |  |  |  |  |  |  |  |  |  |
| n | 20 | 20 | 20 | 20 | 18 | 20 | 20 | 20 | 20 | 19 | 20 |  |
| A | 9 | 21 | 15 | 11 | 14 | 6 | 10 | 12 | 17 | 20 | 14 | 13.5 |
| Ap | 0 | 4 | 1 | 0 | 0 | 1 | 0 | 0 | 2 | 1 | 1 | 0.9 |
| Ar | 7.09 | 12.90 |  | 9.00 |  | 5.33 | 7.68 | 8.32 | 11.08 |  | 8.91 | 8.79 |
| He | 0.800 | 0.850 | 0.850 | 0.950 | 0.889 | 0.750 | 0.900 | 0.750 | 0.900 | 0.842 | 0.900 | 0.853 |
| Ho | 0.829 | 0.955 | 0.933 | 0.908 | 0.930 | 0.805 | 0.865 | 0.859 | 0.932 | 0.954 | 0.878 | 0.895 |
| Fis | 0.036 | **0.113** | 0.091 | -0.048 | 0.046 | 0.07 | -0.041 | 0.13 | 0.035 | **0.121** | -0.025 | 0.048 |

MAD, Madagascar; IND, India; SRI, Sri Lanka; BRU, Brunei; THAI, Thailand; VCM, Vietnam Ca Mau province; VCT, Vietnam Can Tho province; VBL, Vietnam Bac Lieu province; VBT, Vietnam Ben Tre province; PHIL, Philippines; TAI, Taiwan; WAUST, Western Australia; NAUST, Northern Australia; EAUST, Eastern Australia; PAL, Palau; PNG, Papua New Guinea and FIJI, Fiji. F_IS_ values in bold show statistically significant (P<0.05) departures from a Hardy Weinberg equilibrium.
